# Preoperative nutritional status defined by GLIM criteria and postoperative delirium in older patients receiving general anesthesia

**DOI:** 10.3389/fnut.2026.1878893

**Published:** 2026-10-05

**Authors:** Yuan Tian, Ming-Rui Song, Jian-Feng Jiang, Jia-Teng Wu, Yun-Fei Yan, Yan-Yuan Sun

**Affiliations:** 1 Department of Anesthesiology, Shenzhen University General Hospital, Shenzhen, Guangdong, China; 2 Department of Neurology, People’s Hospital of Longhua, Shenzhen, Guangdong, China

**Keywords:** general anesthesia, Global Leadership Initiative on Malnutrition, malnutrition, older patients, postoperative delirium, propensity score matching

## Abstract

**Background:**

Preoperative malnutrition may reflect reduced physiological reserve and increased vulnerability to postoperative delirium (POD) in older surgical patients. However, evidence based on standardized diagnostic criteria remains limited. This study aimed to evaluate the association between Global Leadership Initiative on Malnutrition (GLIM)-defined malnutrition and POD among older patients undergoing surgery under general anesthesia.

**Methods:**

This retrospective observational cohort study included consecutive patients aged ≥65 years who underwent elective or emergency surgery under general anesthesia between January 2022 and December 2025. Preoperative malnutrition was retrospectively classified according to the GLIM framework using available phenotypic and etiologic criteria. Patients with and without GLIM-defined malnutrition were matched 1:2 using propensity score matching. POD documented during hospitalization was identified from Confusion Assessment Method assessments, Confusion Assessment Method for the Intensive Care Unit assessments, or explicit physician diagnoses. The association between GLIM-defined malnutrition and POD was examined using conditional logistic regression stratified by matched set. Subgroup and sensitivity analyses were also performed.

**Results:**

The matched cohort comprised 495 patients, including 165 with GLIM-defined malnutrition and 330 without malnutrition. All propensity score covariates achieved acceptable post-matching balance, with absolute standardized mean differences <0.10. POD occurred in 42 patients with malnutrition (25.5%) and 37 patients without malnutrition (11.2%; *p* < 0.001). GLIM-defined malnutrition was associated with higher odds of POD after multivariable adjustment (adjusted odds ratio, 2.36; 95% confidence interval, 1.36–4.10; *p* = 0.002). No significant interactions were identified across age, surgical category, American Society of Anesthesiologists class, or immediate postoperative intensive care unit admission subgroups. Comparable associations were observed in the unmatched cohort, alternative matched analyses, fixed observation windows, restricted-length-of-stay cohorts, and analyses using alternative POD definitions.

**Conclusion:**

GLIM-defined malnutrition was associated with higher odds of POD during hospitalization in older patients undergoing surgery under general anesthesia. GLIM-based nutritional assessment may provide clinically useful information for perioperative delirium risk stratification.

## 1 Introduction

Postoperative delirium (POD) is a common perioperative neurocognitive complication in older adults, characterized by an acute and fluctuating disturbance in attention, awareness, and cognition (1, 2). It typically occurs during emergence from anesthesia or within the first several postoperative days and is associated with prolonged hospitalization, increased healthcare resource utilization, functional decline, and adverse short-term outcomes (3). A recent large national cohort study of older adults undergoing major noncardiac surgery further demonstrated that POD was strongly associated with mortality, major postoperative complications, and nonhome discharge, highlighting its clinical relevance in contemporary perioperative practice (3–5). Therefore, prevention and early identification of POD remain essential components of perioperative risk management in elderly surgical populations (6).

The pathogenesis of POD is multifactorial and involves both predisposing vulnerability and perioperative precipitating factors. Advanced age, comorbidity burden, surgical stress, inflammatory activation, and reduced physiologic reserve have all been implicated (7, 8). Recent mechanistic studies have emphasized the role of systemic inflammation and secondary neuroinflammation following anesthesia and surgery, while clinical evidence continues to identify vulnerability factors that predispose older patients to acute postoperative brain dysfunction (9, 10). Among these vulnerability domains, nutritional status has received increasing attention. Malnutrition is common among older hospitalized and surgical patients and may compromise immune function, protein synthesis, tissue repair, and tolerance to surgical stress (11, 12). Moreover, malnutrition frequently overlaps with frailty, sarcopenia, anemia, and chronic inflammation, all of which may increase susceptibility to delirium after major surgical exposure (13, 14).

Several recent studies have linked preoperative nutritional measures, including the Geriatric Nutritional Risk Index, Prognostic Nutritional Index, Mini Nutritional Assessment, and inflammatory nutritional markers, with postoperative delirium in older surgical patients (15, 16). However, most evidence has come from single surgical specialties or has relied on screening tools and surrogate laboratory indices rather than standardized diagnostic criteria for malnutrition (17, 18). The Global Leadership Initiative on Malnutrition provides an internationally endorsed framework requiring at least one phenotypic and one etiologic criterion. However, evidence regarding the relationship between GLIM-defined malnutrition and postoperative delirium in older surgical patients remains limited. Therefore, this study aimed to investigate the clinical relevance of preoperative GLIM-defined malnutrition in relation to postoperative delirium among older patients undergoing surgery under general anesthesia.

## 2 Methods

### 2.1 Study design

This retrospective observational cohort study identified consecutive patients aged 65 years or older who underwent elective or emergency surgery under general anesthesia at our institution between January 2022 and December 2025. Patients meeting the GLIM diagnostic criteria constituted the exposure group, and contemporaneous patients who did not meet the GLIM criteria were considered potential controls. Patients were eligible if complete information required to determine preoperative nutritional status and ascertain postoperative delirium during the index hospitalization was available in the electronic medical record. Patients were excluded if they had delirium before surgery; documented dementia or another severe neurocognitive disorder; a major psychiatric illness; a central nervous system disorder likely to substantially interfere with delirium assessment; severe hearing, language, or communication impairment; coma, deep sedation, or mechanical ventilation that precluded valid postoperative cognitive assessment; missing key analytical data; or a postoperative hospital stay of less than 24 h. The study protocol was reviewed and approved by the ethics committee of our hospital. All procedures were conducted in accordance with relevant guidelines and regulations and complied with the Declaration of Helsinki. All data were anonymized before analysis to protect participant privacy.

### 2.2 Preoperative nutritional assessment, GLIM classification, and matching

Preoperative nutritional status was retrospectively determined from the last available clinical and nutritional information recorded within 7 days before surgery. Standardized pre-GLIM screening scores, including the Nutritional Risk Screening 2002, Mini Nutritional Assessment–Short Form, and Malnutrition Universal Screening Tool, were not routinely documented. GLIM status was therefore classified using available medical, nursing, dietary, laboratory, and nutritional records. GLIM-defined malnutrition required at least one phenotypic criterion and one etiologic criterion. The assessable phenotypic criteria were unintentional weight loss of >5% within 6 months and low BMI, defined as <18.5 kg/m^2^ in patients aged 65–69 years and <20.0 kg/m^2^ in those aged ≥70 years. Standardized muscle-mass measurements were not routinely available and were not used. Etiologic criteria included reduced food intake, impaired nutrient assimilation, and disease burden or inflammation. Reduced intake was defined as ≤50% of usual intake during the preceding week or another clinically meaningful reduction lasting >2 weeks. Impaired assimilation was identified from documented gastrointestinal disorders or severe gastrointestinal symptoms likely to affect nutrient digestion or absorption. Disease burden or inflammation was determined from documented acute or chronic inflammatory conditions, with elevated C-reactive protein used as supportive evidence when necessary. Laboratory abnormalities alone were not considered sufficient for diagnosis. Malnutrition severity was graded retrospectively using the available GLIM weight-loss and BMI thresholds. Propensity scores were estimated using multivariable logistic regression including age, sex, elective or emergency surgery, surgical category, American Society of Anesthesiologists physical status, hypertension, diabetes mellitus, coronary artery disease, chronic kidney disease, history of cerebrovascular disease, chronic pulmonary disease, history of malignancy, operative duration, and anesthesia duration. Patients with GLIM-defined malnutrition were matched 1:2 to patients without malnutrition using nearest-neighbor matching without replacement, with a caliper width of 0.20 standard deviations of the logit of the propensity score. Variables constituting or directly reflecting the GLIM classification were not included in the propensity score model. Covariate balance was evaluated using absolute standardized mean differences before and after matching, with values <0.10 considered indicative of acceptable balance. A unique matched-set identifier was retained for matched regression analyses.

### 2.3 Outcome definition and assessment of postoperative delirium

The primary outcome was postoperative delirium documented from the immediate postoperative period through discharge from the index hospitalization. Postoperative delirium was analyzed as a binary outcome. Delirium assessments were retrospectively identified from electronic medical records. CAM or CAM-ICU assessments had been documented in the post-anesthesia care unit, general surgical wards, or intensive care unit by trained clinical staff according to the care setting. However, a mandatory fixed-frequency assessment protocol was not consistently implemented or documented throughout the study period. Assessments were generally performed during routine postoperative care or when an acute change in cognition, attention, consciousness, or behavior was suspected. Patients were classified as having postoperative delirium when a positive CAM or CAM-ICU assessment or an explicit physician-documented diagnosis of delirium was recorded after surgery. Medical records, nursing notes, post-anesthesia care unit records, intensive care records, and consultation notes were reviewed as supporting sources. When no formal CAM assessment was available, CAM criteria were not retrospectively reconstructed from narrative documentation. For patients with multiple records, the earliest documented positive CAM or CAM-ICU assessment or physician diagnosis was considered the first postoperative delirium episode. Delirium subtype was recorded as hypoactive, hyperactive, or mixed only when explicitly documented; otherwise, it was classified as unavailable. Because postoperative length of stay differed between groups, sensitivity analyses were conducted using fixed observation windows of 48 h, 72 h, and 5 postoperative days. Additional analyses were restricted to patients whose postoperative hospital stay was at least as long as the corresponding observation window.

### 2.4 Clinical data collection and quality control

Clinical data were retrospectively extracted from the institutional electronic medical record system. Demographic variables included age, sex, BMI, smoking history, and alcohol consumption history. Baseline comorbidities included hypertension, diabetes mellitus, coronary artery disease, chronic kidney disease, cerebrovascular disease, chronic pulmonary disease, and malignancy. Perioperative variables included elective or emergency surgery, surgical category, operative and anesthesia durations, American Society of Anesthesiologists physical status, intraoperative blood loss, intraoperative blood transfusion, and immediate postoperative intensive care unit admission. Intensive care unit admission was defined as admission directly after surgery. Postoperative length of stay was treated as a descriptive outcome and was not included as a baseline covariate in the primary regression model. Preoperative laboratory variables were obtained from the last available measurement within 7 days before surgery and included hemoglobin, albumin, prealbumin, total protein, blood glucose, serum creatinine, blood urea nitrogen, C-reactive protein, sodium, potassium, and total lymphocyte count. Nutritional information included BMI, weight change within the preceding 6 months, reduced food intake, impaired nutrient assimilation, and disease burden or inflammation. Available preoperative cognitive, frailty, functional, and muscle-related assessments were also reviewed. However, formal cognitive and frailty assessments were sparse and nonuniform, and standardized muscle-mass measurements were unavailable; therefore, these variables were not included in the primary regression model. Data were independently extracted by two investigators using a standardized form. Discrepancies were resolved by review of the original records and discussion, with adjudication by a third investigator when necessary. Missing, implausible, and extreme values were verified against the source records before finalizing the analytical dataset.

### 2.5 Statistical analysis

All statistical analyses were performed using IBM SPSS Statistics, version 26.0 (IBM Corp., Armonk, NY, USA). Continuous variables were summarized as mean ± standard deviation or median with interquartile range and compared using the independent-samples *t* test or Mann–Whitney U test, as appropriate. Categorical variables were expressed as frequencies and percentages and compared using the chi-square test. Covariate balance before and after propensity score matching was assessed using absolute standardized mean differences, with values <0.10 indicating acceptable balance. The association between GLIM-defined malnutrition and postoperative delirium was evaluated using conditional logistic regression, with the matched-set identifier specified as the stratum. The primary fully adjusted model included age, sex, chronic kidney disease, history of cerebrovascular disease, surgical category, American Society of Anesthesiologists class, anesthesia duration per 10-min increase, intraoperative blood transfusion, immediate postoperative intensive care unit admission, and C-reactive protein per 1-mg/L increase. Sequential models were additionally fitted by progressively introducing demographic, comorbidity, surgical, intraoperative, and immediate postoperative variables. Results were reported as odds ratios with 95% confidence intervals. Subgroup analyses were performed according to age, surgical category, American Society of Anesthesiologists class, and immediate postoperative intensive care unit admission. Differences between subgroups were assessed using interaction terms. Sensitivity analyses included multivariable logistic regression in the unmatched cohort, generalized estimating equations with matched sets specified as clusters, and logistic regression with matched-set cluster-robust standard errors. Additional analyses excluded patients admitted directly to the intensive care unit or receiving intraoperative transfusion and replaced anesthesia duration with operative duration. Fixed observation windows of 48 h, 72 h, and 5 days, restricted-length-of-stay cohorts, and alternative postoperative delirium definitions were also examined. All tests were two-sided, with *p* < 0.05 considered statistically significant.

## 3 Results

### 3.1 Study population screening and enrollment

During the study period, 185 older patients undergoing surgery under general anesthesia were initially identified as meeting the GLIM criteria for malnutrition. After application of the predefined eligibility criteria, 20 patients were excluded: 3 with preoperative delirium, 4 with documented dementia or severe neurocognitive disorders, 2 with major psychiatric illness, 3 with central nervous system disorders likely to interfere with delirium assessment, 2 with severe hearing, language, or communication impairment, 4 with missing key analytical data, and 2 with a postoperative hospital stay of less than 24 h. The malnutrition group therefore included 165 patients.

The same eligibility criteria were applied to contemporaneous patients who did not meet the GLIM criteria, leaving 960 eligible patients as potential controls. After 1:2 propensity score matching, 330 patients were selected for the non-malnutrition group. The final matched cohort comprised 495 patients, including 165 with GLIM-defined malnutrition and 330 without malnutrition.

### 3.2 Baseline characteristics and covariate balance after matching

After 1:2 propensity score matching, the matched cohort comprised 165 patients with GLIM-defined malnutrition and 330 patients without malnutrition. All prespecified variables included in the propensity score model had absolute standardized mean differences below 0.10 after matching, indicating acceptable balance in age, sex, elective or emergency surgery, surgical category, ASA class, major comorbidities, and operative and anesthesia durations. The corresponding covariate distributions before and after matching are presented in Supplementary Table S1. As expected, variables directly related to nutritional status remained different between the two groups. Compared with patients without malnutrition, patients with GLIM-defined malnutrition had a lower BMI, greater recent weight loss, and lower hemoglobin, albumin, prealbumin, total protein, and total lymphocyte count values. Blood urea nitrogen and C-reactive protein levels were higher in the malnutrition group, whereas the between-group difference in serum creatinine did not reach statistical significance. Median postoperative length of hospital stay was 12 days (interquartile range, 9–16 days) in the malnutrition group and 10 days (interquartile range, 8–14 days) in the non-malnutrition group (*p* = 0.008) (Table 1). Among the 165 patients meeting the GLIM diagnostic criteria, 112 (67.9%) were classified as having moderate malnutrition and 53 (32.1%) as having severe malnutrition. The operational definitions and distribution of the individual phenotypic and etiologic GLIM components are provided in Supplementary Table S2.

**Table 1:** Characteristics of patients with and without GLIM-defined malnutrition after 1:2 matching.

Variable	GLIM-defined malnutrition group (*n* = 165)	Non-malnutrition group (*n* = 330)	*p* value	Absolute SMD
Demographic characteristics				
Age, years	73.8 ± 6.1	73.4 ± 6.0	0.495	0.066
Male sex, *n* (%)	96 (58.2)	188 (57.0)	0.81	0.025
BMI, kg/m^2^	20.3 ± 2.1	23.8 ± 2.6	<0.001	1.481
Smoking history, *n* (%)	54 (32.7)	101 (30.6)	0.633	0.046
Alcohol consumption history, *n* (%)	38 (23.0)	69 (20.9)	0.597	0.051
Comorbidities				
Hypertension, *n* (%)	98 (59.4)	189 (57.3)	0.658	0.043
Diabetes mellitus, *n* (%)	47 (28.5)	86 (26.1)	0.573	0.054
Coronary artery disease, *n* (%)	39 (23.6)	70 (21.2)	0.548	0.058
Chronic kidney disease, *n* (%)	21 (12.7)	32 (9.7)	0.306	0.096
History of cerebrovascular disease, *n* (%)	26 (15.8)	45 (13.6)	0.517	0.06
Chronic pulmonary disease, *n* (%)	29 (17.6)	52 (15.8)	0.611	0.049
History of malignancy, *n* (%)	18 (10.9)	31 (9.4)	0.602	0.05
Perioperative characteristics				
Surgical category, *n* (%)			0.996	0.049
Orthopedic surgery	71 (43.0)	145 (43.9)		0.018
Gastrointestinal surgery	32 (19.4)	62 (18.8)		0.015
Hepatobiliary/pancreatic surgery	21 (12.7)	40 (12.1)		0.018
Urologic surgery	18 (10.9)	34 (10.3)		0.02
Thoracic surgery	13 (7.9)	25 (7.6)		0.011
Other surgery	10 (6.1)	24 (7.3)		0.049
Operative duration, min	154.7 ± 46.3	151.2 ± 44.8	0.426	0.077
Anesthesia duration, min	188.5 ± 51.6	184.3 ± 49.7	0.387	0.083
ASA class III–IV, *n* (%)	102 (61.8)	198 (60.0)	0.699	0.037
Intraoperative blood loss, mL	220 (150–350)	200 (140–330)	0.242	0.138
Intraoperative blood transfusion, *n* (%)	27 (16.4)	46 (13.9)	0.455	0.068
Immediate postoperative ICU admission, *n* (%)	31 (18.8)	55 (16.7)	0.562	0.056
Laboratory and nutrition-related variables				
Hemoglobin, g/L	108.6 ± 16.8	121.4 ± 15.2	<0.001	0.799
Albumin, g/L	31.8 ± 3.9	38.6 ± 4.1	<0.001	1.699
Prealbumin, mg/L	158.4 ± 41.7	221.6 ± 48.5	<0.001	1.397
Total protein, g/L	60.9 ± 6.7	67.8 ± 6.4	<0.001	1.053
Blood glucose, mmol/L	6.4 ± 1.8	6.1 ± 1.6	0.066	0.176
Serum creatinine, μmol/L	88.5 ± 27.4	84.1 ± 24.9	0.079	0.168
Blood urea nitrogen, mmol/L	7.6 ± 2.8	6.9 ± 2.4	0.004	0.268
C-reactive protein, mg/L	10.8 (5.6–21.4)	7.2 (3.9–15.8)	0.001	0.347
Sodium, mmol/L	138.2 ± 3.4	138.8 ± 3.1	0.049	0.184
Potassium, mmol/L	4.10 ± 0.43	4.16 ± 0.39	0.122	0.146
Total lymphocyte count, ×10^9^/L	1.21 ± 0.42	1.58 ± 0.47	<0.001	0.83
Recent weight loss within 6 months, %	6.8 (4.5–9.7)	1.9 (0.8–3.4)	<0.001	1.608
Postoperative descriptive outcome				
Length of hospital stay, days	12 (9–16)	10 (8–14)	0.008	Not applicable

### 3.3 Incidence, timing, and ascertainment of postoperative delirium

Postoperative delirium during hospitalization was documented in 42 of 165 patients (25.5%) with GLIM-defined malnutrition and 37 of 330 patients (11.2%) without malnutrition (*p* < 0.001). Among patients who developed postoperative delirium, the first documented episode occurred within 48 postoperative hours in 29 of 42 patients (69.0%) in the malnutrition group and 16 of 37 patients (43.2%) in the non-malnutrition group. A further 10 patients (23.8%) and 16 patients (43.2%), respectively, had their first documented episode more than 48 h after surgery through postoperative day 5. The timing distribution did not differ significantly between the groups (*p* = 0.069) (Table 2). At least one CAM assessment was documented in 432 of 495 patients (87.3%), and a structured CAM record was available in 301 patients (60.8%). Among the 79 patients with postoperative delirium, 58 (73.4%) had a positive CAM or CAM-ICU assessment and 52 (65.8%) had a documented physician diagnosis; these ascertainment sources were not mutually exclusive. Hypoactive delirium was explicitly documented in 29 patients (36.7%), whereas the motor subtype could not be reliably classified in 18 patients (22.8%). Additional information on assessment locations and ascertainment sources is presented in Supplementary Table S3.

**Table 2:** Incidence and timing of the first documented episode of postoperative delirium during hospitalization.

Variable	GLIM-defined malnutrition group (*n* = 165)	Non-malnutrition group (*n* = 330)	Test statistic	*p* value
Occurrence of postoperative delirium during hospitalization, *n* (%)				
Postoperative delirium	42 (25.5)	37 (11.2)	Chi-square = 16.64	<0.001
No postoperative delirium	123 (74.5)	293 (88.8)		
Timing of the first documented episode of postoperative delirium, *n* (%)				
Within 48 postoperative hours	29 (69.0)	16 (43.2)	Chi-square = 5.35	0.069
More than 48 h through postoperative day 5	10 (23.8)	16 (43.2)		
After postoperative day 5	3 (7.1)	5 (13.5)		

GLIM, Global Leadership Initiative on Malnutrition; CAM, Confusion Assessment Method; CAM-ICU, Confusion Assessment Method for the Intensive Care Unit.

### 3.4 Characteristics of patients with and without postoperative delirium

Among the 495 matched patients, 79 developed postoperative delirium and 416 did not. Patients with postoperative delirium were older than those without postoperative delirium (76.4 ± 6.4 vs. 73.0 ± 5.9 years, *p* < 0.001). Chronic kidney disease and a history of cerebrovascular disease were more frequent in the postoperative delirium group. Several perioperative characteristics also differed between the groups. Patients with postoperative delirium had longer anesthesia duration (208.9 ± 55.2 vs. 181.3 ± 48.3 min, *p* < 0.001), a higher frequency of ASA class III–IV (74.7% vs. 57.9%, *p* = 0.005), intraoperative blood transfusion (25.3% vs. 12.7%, *p* = 0.004), and immediate postoperative ICU admission (29.1% vs. 15.1%, *p* = 0.003). Surgical-category distributions were similar between the groups (*p* = 0.719). The postoperative delirium group also had lower BMI and lower hemoglobin, albumin, prealbumin, total protein, sodium, and total lymphocyte count values, together with higher blood urea nitrogen, serum creatinine, and C-reactive protein levels. GLIM-defined malnutrition was present in 42 of 79 patients (53.2%) with postoperative delirium and 123 of 416 patients (29.6%) without postoperative delirium (*p* < 0.001). Postoperative length of hospital stay was longer among patients with postoperative delirium; this variable was treated as a postoperative descriptive outcome rather than a baseline predictor (Table 3).

**Table 3:** Clinical, perioperative, laboratory, nutritional, and postoperative characteristics of patients with and without postoperative delirium.

Variable	Postoperative delirium group (*n* = 79)	Non-postoperative delirium group (*n* = 416)	Test statistic	*p* value
Demographic characteristics				
Age, years	76.4 ± 6.4	73.0 ± 5.9	*t* = 4.63	<0.001
Male sex, *n* (%)	52 (65.8)	232 (55.8)	Chi-square = 2.74	0.098
BMI, kg/m^2^	20.7 ± 2.4	23.0 ± 2.9	*t* = −6.63	<0.001
Smoking history, *n* (%)	30 (38.0)	125 (30.0)	Chi-square = 1.94	0.164
Alcohol consumption history, *n* (%)	20 (25.3)	87 (20.9)	Chi-square = 0.76	0.383
Comorbidities				
Hypertension, *n* (%)	52 (65.8)	235 (56.5)	Chi-square = 2.37	0.123
Diabetes mellitus, *n* (%)	26 (32.9)	107 (25.7)	Chi-square = 1.75	0.186
Coronary artery disease, *n* (%)	22 (27.8)	87 (20.9)	Chi-square = 1.86	0.173
Chronic kidney disease, *n* (%)	14 (17.7)	39 (9.4)	Chi-square = 4.84	0.028
History of cerebrovascular disease, *n* (%)	18 (22.8)	53 (12.7)	Chi-square = 5.45	0.02
Chronic pulmonary disease, *n* (%)	17 (21.5)	64 (15.4)	Chi-square = 1.83	0.177
History of malignancy, *n* (%)	10 (12.7)	39 (9.4)	Chi-square = 0.80	0.37
Perioperative characteristics				
Surgical category, *n* (%)			Chi-square = 2.88	0.719
Orthopedic surgery	40 (50.6)	176 (42.3)		
Gastrointestinal surgery	14 (17.7)	80 (19.2)		
Hepatobiliary/pancreatic surgery	8 (10.1)	53 (12.7)		
Urologic surgery	6 (7.6)	46 (11.1)		
Thoracic surgery	7 (8.9)	31 (7.5)		
Other surgery	4 (5.1)	30 (7.2)		
Operative duration, min	171.3 ± 49.8	148.8 ± 43.6	*t* = 4.11	<0.001
Anesthesia duration, min	208.9 ± 55.2	181.3 ± 48.3	*t* = 4.55	<0.001
ASA class III–IV, *n* (%)	59 (74.7)	241 (57.9)	Chi-square = 7.80	0.005
Intraoperative blood loss, mL	286 ± 145	198 ± 132	*t* = 5.35	<0.001
Intraoperative blood transfusion, *n* (%)	20 (25.3)	53 (12.7)	Chi-square = 8.35	0.004
Immediate postoperative ICU admission, *n* (%)	23 (29.1)	63 (15.1)	Chi-square = 9.03	0.003
Laboratory and nutrition-related variables				
Hemoglobin, g/L	104.2 ± 17.5	119.6 ± 15.7	*t* = −7.84	<0.001
Albumin, g/L	31.2 ± 4.4	37.4 ± 4.5	*t* = −11.27	<0.001
Prealbumin, mg/L	152.8 ± 45.6	209.6 ± 52.0	*t* = −9.07	<0.001
Total protein, g/L	59.8 ± 7.4	66.7 ± 6.7	*t* = −8.25	<0.001
Blood glucose, mmol/L	6.5 ± 1.9	6.1 ± 1.6	*t* = 1.97	0.049
Serum creatinine, μmol/L	92.7 ± 29.8	84.2 ± 24.8	*t* = 2.70	0.007
Blood urea nitrogen, mmol/L	8.3 ± 3.1	6.8 ± 2.3	*t* = 5.00	<0.001
C-reactive protein, mg/L	14.6 ± 8.9	8.1 ± 6.4	*t* = 7.72	<0.001
Sodium, mmol/L	137.9 ± 3.7	138.8 ± 3.2	*t* = −2.23	0.026
Potassium, mmol/L	4.08 ± 0.46	4.15 ± 0.40	*t* = −1.39	0.165
Total lymphocyte count, ×10^9^/L	1.13 ± 0.38	1.52 ± 0.48	*t* = −6.82	<0.001
Recent weight loss within 6 months, %	6.5 ± 3.4	2.8 ± 2.1	*t* = 12.81	<0.001
GLIM-defined malnutrition, *n* (%)	42 (53.2)	123 (29.6)	Chi-square = 16.64	<0.001
Postoperative descriptive outcome				
Length of hospital stay, days	14.8 ± 5.1	10.9 ± 4.3	*t* = 7.16	<0.001

GLIM, Global Leadership Initiative on Malnutrition; BMI, body mass index; ASA, American Society of Anesthesiologists; ICU, intensive care unit.

### 3.5 Primary matched-set regression analysis

In the conditional logistic regression model stratified by matched-set identifier, GLIM-defined malnutrition was associated with higher odds of postoperative delirium after adjustment for age, sex, chronic kidney disease, history of cerebrovascular disease, surgical category, ASA class, anesthesia duration, intraoperative blood transfusion, immediate postoperative ICU admission, and C-reactive protein level (adjusted OR = 2.36, 95% CI: 1.36–4.10, *p* = 0.002). Older age was associated with higher odds of postoperative delirium (adjusted OR per 1-year increase = 1.08, 95% CI: 1.03–1.13, *p* = 0.001). Other variables associated with postoperative delirium included a history of cerebrovascular disease (adjusted OR = 2.01, 95% CI: 1.06–3.82, *p* = 0.033), longer anesthesia duration (adjusted OR per 10-min increase = 1.07, 95% CI: 1.02–1.12, *p* = 0.005), intraoperative blood transfusion (adjusted OR = 1.91, 95% CI: 1.01–3.62, *p* = 0.047), immediate postoperative ICU admission (adjusted OR = 2.09, 95% CI: 1.12–3.90, *p* = 0.021), and higher C-reactive protein level (adjusted OR per 1-mg/L increase = 1.05, 95% CI: 1.02–1.08, *p* = 0.001). The confidence intervals for chronic kidney disease and ASA class III–IV included the null value in the fully adjusted model (Table 4).

**Table 4:** Selected estimates from the primary matched-set conditional logistic regression analysis of postoperative delirium during hospitalization.

Variable	Adjusted OR	95% CI	*p* value
GLIM-defined malnutrition	2.36	1.36–4.10	0.002
Age, per 1-year increase	1.08	1.03–1.13	0.001
Chronic kidney disease	1.86	0.98–3.55	0.059
History of cerebrovascular disease	2.01	1.06–3.82	0.033
ASA class III–IV	1.75	0.99–3.11	0.055
Anesthesia duration, per 10-min increase	1.07	1.02–1.12	0.005
Intraoperative blood transfusion	1.91	1.01–3.62	0.047
Immediate postoperative ICU admission	2.09	1.12–3.90	0.021
C-reactive protein, per 1-mg/L increase	1.05	1.02–1.08	0.001

The fully adjusted model additionally included sex and surgical category; their individual estimates are not displayed because the table presents selected clinically relevant estimates.

GLIM, Global Leadership Initiative on Malnutrition; OR, odds ratio; CI, confidence interval; ASA, American Society of Anesthesiologists; ICU, intensive care unit.

### 3.6 Subgroup analyses

The adjusted odds ratios for the association between GLIM-defined malnutrition and postoperative delirium were greater than 1.0 across all examined subgroups. The estimates were 2.08 (95% CI: 1.02–4.24) among patients aged 65–74 years and 2.59 (95% CI: 1.26–5.32) among those aged 75 years or older. Similar estimates were observed in orthopedic and non-orthopedic surgical subgroups. Among patients with ASA class I–II, the adjusted OR was 2.12 (95% CI: 0.95–4.72), whereas the corresponding estimate among patients with ASA class III–IV was 2.41 (95% CI: 1.25–4.65). The adjusted ORs were 2.27 (95% CI: 1.26–4.10) among patients without immediate postoperative ICU admission and 2.51 (95% CI: 0.94–6.72) among those admitted to the ICU. No statistically significant interaction was identified by age, surgical category, ASA class, or immediate postoperative ICU admission status (all *p* for interaction > 0.05) (Table 5).

**Table 5:** Subgroup analyses of the association between GLIM-defined malnutrition and postoperative delirium.

Subgroup	*n*	Adjusted OR	95% CI	*p* value	*p* for interaction
Age					0.438
65–74 years	258	2.08	1.02–4.24	0.044	
75 years or older	237	2.59	1.26–5.32	0.01	
Surgical category					0.612
Orthopedic surgery	216	2.49	1.20–5.17	0.014	
Non-orthopedic surgery	279	2.22	1.12–4.40	0.022	
ASA class					0.724
ASA class I–II	195	2.12	0.95–4.72	0.066	
ASA class III–IV	300	2.41	1.25–4.65	0.009	
Immediate postoperative ICU admission					0.793
No	409	2.27	1.26–4.10	0.006	
Yes	86	2.51	0.94–6.72	0.067	

### 3.7 Sensitivity analyses

The association between GLIM-defined malnutrition and postoperative delirium was similar across alternative analytical approaches. In the unmatched cohort, the adjusted OR was 2.53 (95% CI: 1.63–3.92, *p* < 0.001). The estimate obtained using generalized estimating equations was 2.39 (95% CI: 1.40–4.08, *p* = 0.001). Results were also similar after excluding patients admitted to the ICU (adjusted OR = 2.24, 95% CI: 1.22–4.11), excluding patients who received an intraoperative blood transfusion (adjusted OR = 2.17, 95% CI: 1.23–3.83), and replacing anesthesia duration with operative duration (adjusted OR = 2.34, 95% CI: 1.35–4.05) (Table 6). Analyses using fixed postoperative observation windows produced estimates in the same direction. The adjusted ORs were 3.01 (95% CI: 1.52–5.96) for postoperative delirium documented within 48 h, 2.74 (95% CI: 1.49–5.04) within 72 h, and 2.48 (95% CI: 1.40–4.41) through postoperative day 5. Restricting the analyses to patients hospitalized for at least 48 h, 72 h, or 5 days yielded adjusted ORs ranging from 2.29 to 2.34. Alternative outcome definitions restricted to structured CAM-positive, physician-confirmed, or CAM-positive or specialist-confirmed delirium also produced comparable estimates (Supplementary Table S4). Formal preoperative cognitive assessments were available for only a small proportion of the cohort: MMSE scores were available in 26 patients (5.3%), MoCA scores in 12 patients (2.4%), and Mini-Cog assessments in 9 patients (1.8%). A formal Clinical Frailty Scale score was available in 18 patients (3.6%), and no standardized muscle-mass measurements were available. Because these data were sparse and were not collected uniformly, cognitive status, frailty, and muscle mass were not included in the primary regression model (Supplementary Tables S5, S6).

**Table 6:** Sensitivity analyses of the association between GLIM-defined malnutrition and postoperative delirium.

Analysis	Sample size	Adjusted OR	95% CI	*p* value
Unmatched cohort with multivariable adjustment	1,125	2.53	1.63–3.92	<0.001
Primary matched-set conditional logistic regression	495	2.36	1.36–4.10	0.002
Alternative matched analysis using GEE	495	2.39	1.40–4.08	0.001
Excluding patients admitted to the ICU	409	2.24	1.22–4.11	0.009
Excluding patients receiving intraoperative blood transfusion	422	2.17	1.23–3.83	0.008
Operative duration replacing anesthesia duration	495	2.34	1.35–4.05	0.002

## 4 Discussion

This retrospective propensity score–matched cohort study showed that GLIM-defined malnutrition was associated with higher odds of postoperative delirium among older patients undergoing a broad range of surgical procedures under general anesthesia. The principal contribution of this study is the evaluation of nutritional vulnerability using the consensus-based GLIM framework, which integrates phenotypic and etiologic information, rather than relying exclusively on isolated laboratory indices or screening scores (19, 20). Previous studies have primarily examined the prognostic nutritional index, geriatric nutritional risk index, Mini Nutritional Assessment–Short Form, or Nutritional Risk Screening 2002, often within selected surgical populations (21, 22). By combining 1:2 propensity score matching with matched-set conditional logistic regression, subgroup evaluation, fixed postoperative observation windows, and alternative delirium definitions, the present analysis extends this evidence to a heterogeneous older surgical population. The findings support GLIM-defined malnutrition as a clinically informative marker of perioperative vulnerability that may complement established assessments of age, comorbidity, surgical burden, and physiological reserve (23). Because the study was observational, the results do not establish that malnutrition causes delirium or that nutritional intervention would necessarily reduce its occurrence. Nevertheless, preoperative identification of GLIM-defined malnutrition may help clinicians recognize patients who warrant closer perioperative surveillance and more systematic implementation of established multicomponent delirium-prevention measures (24).

Postoperative delirium was documented in 25.5% of patients with GLIM-defined malnutrition and 11.2% of those without malnutrition, while the matched-set analysis demonstrated an approximately twofold difference in adjusted odds. This finding is broadly consistent with previous observational studies linking poorer preoperative nutritional status, assessed using the prognostic nutritional index, geriatric nutritional risk index, Mini Nutritional Assessment–Short Form, Nutritional Risk Screening 2002, or anthropometric measures, to postoperative delirium in older surgical populations (22, 25). In the present cohort, patients with GLIM-defined malnutrition had lower BMI, greater recent weight loss, and lower concentrations of hemoglobin, albumin, prealbumin, total protein, and circulating lymphocytes, together with higher blood urea nitrogen and C-reactive protein levels. These accompanying characteristics indicate that the GLIM classification identified patients with concurrent deficits in energy and protein reserves, impaired hematologic and immune–nutritional status, and greater systemic disease burden. Such vulnerability may be relevant to delirium because the aging brain has reduced physiological reserve and may be less able to maintain cerebral homeostasis during perioperative inflammation, fasting, fluid shifts, blood loss, anesthetic exposure, and metabolic disturbance (26, 27). Reduced nutrient availability may also impair neurotransmitter synthesis, antioxidant capacity, and cellular repair, whereas systemic inflammation may promote endothelial dysfunction, blood–brain barrier permeability, and neuroinflammatory signaling (28, 29). These mechanisms remain biologically plausible rather than directly demonstrated by the present data. Moreover, C-reactive protein should not be interpreted as an entirely independent pathway because inflammatory disease burden formed part of the GLIM etiologic assessment (30). Differences between the present findings and studies reporting no association between nutritional risk and early delirium may reflect variation in patient selection, nutritional instruments, delirium definitions, observation periods, surgical severity, and adjustment for baseline cognition (31). Taken together, the results suggest that the observed association reflects a complex nutritional–inflammatory vulnerability phenotype rather than the influence of any single laboratory or anthropometric measure.

The temporal analyses indicated that postoperative delirium was predominantly an early postoperative event, particularly among patients with GLIM-defined malnutrition. Among malnourished patients who developed delirium, 69.0% had their first documented episode within 48 h after surgery, compared with 43.2% of delirium cases in the non-malnutrition group. Although the overall timing distribution did not reach statistical significance, this pattern suggests that the period immediately following anesthesia and surgery may be especially relevant for nutritionally vulnerable patients, when inflammatory activation, pain, sleep disruption, hemodynamic instability, medication exposure, and metabolic disturbances are most pronounced (32, 33). The association between GLIM-defined malnutrition and delirium remained evident when the outcome was restricted to 48 h, 72 h, and 5 postoperative days, with adjusted odds ratios of 3.01, 2.74, and 2.48, respectively. Similar estimates were obtained after restricting analyses to patients whose hospitalization was sufficiently long to cover each observation window, indicating that the principal result was unlikely to be attributable solely to the longer postoperative stay in the malnutrition group. The findings were also consistent in the unmatched cohort, in generalized estimating equation analyses, after exclusion of patients admitted directly to the intensive care unit or receiving intraoperative transfusion, and when operative duration replaced anesthesia duration. Subgroup estimates remained above unity across age groups, orthopedic and non-orthopedic procedures, ASA classes, and immediate postoperative ICU status, with no statistically significant interaction. These results do not establish equivalent associations across all subgroups, particularly where confidence intervals were wide, but they provide no clear evidence of substantial effect heterogeneity. The accompanying associations with older age, cerebrovascular disease, longer anesthesia duration, transfusion, and immediate ICU admission further support a multifactorial model in which baseline susceptibility and perioperative stress jointly shape delirium occurrence (34, 35). From a practical perspective, the concentration of events within the first 48–72 h supports prioritizing structured cognitive observation during this period in older patients with preoperative nutritional vulnerability (36, 37).

The present findings can be compared most directly with three recent studies. De la Varga-Martínez et al. evaluated 2,442 surgical patients across 43 Spanish hospitals and reported an early postoperative delirium incidence of 3.93%, with older age and preoperative cognitive impairment among the principal associated factors (38). Our study similarly found an association between increasing age and postoperative delirium but documented a higher overall incidence, particularly among patients with GLIM-defined malnutrition. This difference may reflect variation in case mix, the restriction of our cohort to patients aged ≥65 years, and our assessment of delirium throughout hospitalization rather than only through postoperative day 2. Unlike the DELPO study, our analysis focused specifically on nutritional vulnerability and showed that GLIM-defined malnutrition remained associated with delirium after accounting for demographic, comorbidity, and perioperative covariates. Luo et al. synthesized 50 cohort studies involving 1,182,719 patients undergoing spine surgery and reported associations of postoperative delirium with older age, cerebrovascular disease, kidney disease, ICU admission, ASA class >II, and blood transfusion (39). These pooled findings are broadly consistent with our results for age, previous cerebrovascular disease, intraoperative transfusion, and immediate postoperative ICU admission. However, chronic kidney disease and ASA class III–IV showed only directionally positive estimates in our fully adjusted model, with confidence intervals including the null value. This difference may arise from the broader surgical composition of our cohort, differences in covariate adjustment, and the procedure-specific population included in the meta-analysis. Szczupak et al. retrospectively studied 408 cardiac and neurosurgical ICU patients and observed more frequent or severe delirium among patients with elevated CRP measured on postoperative days 1 and 2 (40). Our finding involved preoperative CRP measured within 7 days before surgery and therefore reflects baseline inflammatory burden rather than the acute postoperative response. Moreover, CRP was used as supportive evidence for the inflammatory etiologic component of GLIM classification; its association should therefore not be interpreted as independent of nutritional-inflammatory vulnerability. Collectively, these comparisons support a multidimensional model in which nutritional status, neurological reserve, systemic inflammation, and perioperative stress are jointly associated with postoperative delirium.

Several limitations should be acknowledged. First, the retrospective single-center design may have introduced selection bias and residual confounding despite propensity score matching, multivariable adjustment, and sensitivity analyses. Delirium assessment was based on routine clinical documentation rather than a uniformly scheduled protocol, which may have resulted in under-recognition of transient or hypoactive episodes. Differential observation time also cannot be completely excluded, although fixed-window and length-of-stay-restricted analyses produced consistent findings. Second, standardized assessments of baseline cognition, frailty, sarcopenia, and muscle mass were unavailable or inconsistently documented. Residual confounding may therefore remain, and some patients with reduced muscle mass may have been missed by the Global Leadership Initiative on Malnutrition classification. Third, malnutrition status was reconstructed retrospectively because standardized nutritional screening was not routinely performed. Fourth, only in-hospital delirium was evaluated, and longer-term cognitive outcomes were unavailable. Finally, the observational design does not permit causal inference. Future prospective multicenter studies should use standardized nutritional, cognitive, frailty, muscle-mass, and delirium assessments, include longer-term follow-up, and evaluate whether nutritional optimization combined with multicomponent delirium-prevention strategies improves postoperative cognitive outcomes.

## 5 Conclusion

In conclusion, GLIM-defined malnutrition was associated with higher odds of postoperative delirium during hospitalization among older patients undergoing surgery under general anesthesia. This association was consistent across alternative analytical approaches, clinically relevant subgroups, fixed postoperative observation windows, and alternative delirium definitions. These findings suggest that GLIM-based nutritional assessment may provide useful information for perioperative delirium risk stratification. Prospective studies are warranted to confirm this association and to determine whether targeted nutritional optimization, when integrated into multicomponent perioperative care, can improve postoperative cognitive outcomes.

## Data Availability

The raw data supporting the conclusions of this article will be made available by the authors, without undue reservation.
