# Supplementary material for: Preoperative nutritional status defined by GLIM criteria and postoperative delirium in older patients receiving general anesthesia
Source: Front Nutr. 2026 Oct 5;13:1878893. doi: 10.3389/fnut.2026.1878893 (PMC13677495; doi:10.3389/fnut.2026.1878893)
Supplement: Supplementary file 1 [file Table_1.docx]

Supplementary Table S1. Covariate balance before and after 1:2 propensity score matching

| Variable | Before matching: GLIM malnutrition group, n = 165 | Before matching: non-malnutrition group, n = 960 | Absolute SMD before matching | After matching: GLIM malnutrition group, n = 165 | After matching: non-malnutrition group, n = 330 | Absolute SMD after matching |
| --- | --- | --- | --- | --- | --- | --- |
| Age, years | 73.8 (6.1) | 72.0 (6.4) | 0.288 | 73.8 (6.1) | 73.4 (6.0) | 0.066 |
| Male sex, n (%) | 96 (58.2) | 515 (53.6) | 0.091 | 96 (58.2) | 188 (57.0) | 0.025 |
| Elective surgery, n (%) | 137 (83.0) | 856 (89.2) | 0.178 | 137 (83.0) | 277 (83.9) | 0.024 |
| Emergency surgery, n (%) | 28 (17.0) | 104 (10.8) | 0.178 | 28 (17.0) | 53 (16.1) | 0.024 |
| Orthopedic surgery, n (%) | 71 (43.0) | 390 (40.6) | 0.049 | 71 (43.0) | 145 (43.9) | 0.018 |
| Gastrointestinal surgery, n (%) | 32 (19.4) | 190 (19.8) | 0.010 | 32 (19.4) | 62 (18.8) | 0.015 |
| Hepatobiliary or pancreatic surgery, n (%) | 21 (12.7) | 125 (13.0) | 0.009 | 21 (12.7) | 40 (12.1) | 0.018 |
| Urologic surgery, n (%) | 18 (10.9) | 110 (11.5) | 0.017 | 18 (10.9) | 34 (10.3) | 0.020 |
| Thoracic surgery, n (%) | 13 (7.9) | 70 (7.3) | 0.022 | 13 (7.9) | 25 (7.6) | 0.011 |
| Other surgery, n (%) | 10 (6.1) | 75 (7.8) | 0.069 | 10 (6.1) | 24 (7.3) | 0.049 |
| ASA class III-IV, n (%) | 102 (61.8) | 505 (52.6) | 0.187 | 102 (61.8) | 198 (60.0) | 0.037 |
| Hypertension, n (%) | 98 (59.4) | 515 (53.6) | 0.116 | 98 (59.4) | 189 (57.3) | 0.043 |
| Diabetes mellitus, n (%) | 47 (28.5) | 220 (22.9) | 0.128 | 47 (28.5) | 86 (26.1) | 0.054 |
| Coronary artery disease, n (%) | 39 (23.6) | 175 (18.2) | 0.133 | 39 (23.6) | 70 (21.2) | 0.058 |
| Chronic kidney disease, n (%) | 21 (12.7) | 70 (7.3) | 0.182 | 21 (12.7) | 32 (9.7) | 0.096 |
| History of cerebrovascular disease, n (%) | 26 (15.8) | 95 (9.9) | 0.176 | 26 (15.8) | 45 (13.6) | 0.060 |
| Chronic pulmonary disease, n (%) | 29 (17.6) | 130 (13.5) | 0.111 | 29 (17.6) | 52 (15.8) | 0.049 |
| History of malignancy, n (%) | 18 (10.9) | 70 (7.3) | 0.126 | 18 (10.9) | 31 (9.4) | 0.050 |
| Operative duration, min | 154.7 (46.3) | 143.0 (45.5) | 0.255 | 154.7 (46.3) | 151.2 (44.8) | 0.077 |
| Anesthesia duration, min | 188.5 (51.6) | 174.0 (49.8) | 0.286 | 188.5 (51.6) | 184.3 (49.7) | 0.083 |

Abbreviations: GLIM, Global Leadership Initiative on Malnutrition; SMD, standardized mean difference; ASA, American Society of Anesthesiologists.

Supplementary Table S2. Operational definitions and components of the GLIM diagnosis in the matched cohort

| GLIM domain | Criterion | Operational definition used | Data source | Available, n/N (%) | Patients meeting criterion, n (%) |
| --- | --- | --- | --- | --- | --- |
| Nutritional risk screening | Standardized screening tool | NRS-2002, MNA-SF, MUST, or another standardized screening score was not routinely documented. GLIM assessment was performed using available preoperative nutritional information. | Preoperative medical and nursing records | 0/495 (0.0) | Not applicable |
| Nutritional risk screening | Screening timing | Nutritional information was extracted from the last preoperative assessment performed within 7 days before surgery. | Electronic medical records | 495/495 (100.0) | Not applicable |
| Phenotypic criterion | Unintentional weight loss | Unintentional weight loss greater than 5 percent during the 6 months before surgery. Weight loss beyond 6 months could not be evaluated consistently. | Preoperative history, nursing admission records, and nutritional assessment records | 495/495 (100.0) | 176/495 (35.6) |
| Phenotypic criterion | Low BMI | For the Asian population, low BMI was defined as less than 18.5 kg/m2 in patients aged 65 to 69 years and less than 20.0 kg/m2 in patients aged 70 years or older. | Height and weight measured during the preoperative admission | 495/495 (100.0) | 123/495 (24.8) |
| Phenotypic criterion | Reduced muscle mass | CT-derived skeletal muscle index, bioelectrical impedance analysis, dual-energy X-ray absorptiometry, calf circumference, and other validated muscle-mass measurements were not routinely performed. | Imaging, nutritional, rehabilitation, and nursing records | 0/495 (0.0) | Not assessed |
| Etiologic criterion | Reduced food intake | Intake of 50 percent or less of usual intake during the preceding week, or any clinically meaningful reduction in intake lasting more than 2 weeks. | Dietitian notes, medical history, nursing dietary records, and nutrition-related admission records | 432/495 (87.3) | 126/495 (25.5) |
| Etiologic criterion | Reduced nutrient assimilation | A documented chronic gastrointestinal condition or severe gastrointestinal symptoms considered likely to impair nutrient digestion, absorption, or assimilation. | Medical history, gastrointestinal diagnoses, operative diagnoses, and nutrition-related records | 495/495 (100.0) | 39/495 (7.9) |
| Etiologic criterion | Disease burden or inflammation | Acute or chronic disease, infection, injury, or malignancy considered to have clinically relevant inflammatory activity. CRP above the local laboratory reference limit was used as supportive evidence when the inflammatory contribution was uncertain. | Admission diagnoses, comorbidity records, clinical notes, and preoperative CRP | 495/495 (100.0) | 241/495 (48.7) |
| Final diagnosis | GLIM-defined malnutrition | Presence of at least one phenotypic criterion and at least one etiologic criterion. Low albumin, prealbumin, hemoglobin, or lymphocyte count alone was not considered sufficient for the GLIM diagnosis. | Integrated review of all available nutritional and clinical records | 495/495 (100.0) | 165/495 (33.3) |
| Severity grading | Moderate malnutrition | Moderate severity was defined by weight loss of 5 to 10 percent within 6 months, weight loss of 10 to 20 percent beyond 6 months when available, BMI less than 20 kg/m2 for patients younger than 70 years, or BMI less than 22 kg/m2 for patients aged 70 years or older, without meeting a severe threshold. | Phenotypic GLIM information | 165/165 (100.0) | 112/165 (67.9) |
| Severity grading | Severe malnutrition | Severe severity was defined by weight loss greater than 10 percent within 6 months, weight loss greater than 20 percent beyond 6 months when available, BMI less than 18.5 kg/m2 for patients younger than 70 years, or BMI less than 20 kg/m2 for patients aged 70 years or older. | Phenotypic GLIM information | 165/165 (100.0) | 53/165 (32.1) |

Abbreviations: GLIM, Global Leadership Initiative on Malnutrition; BMI, body mass index; CRP, C-reactive protein; CT, computed tomography; NRS-2002, Nutritional Risk Screening 2002; MNA-SF, Mini Nutritional Assessment Short Form; MUST, Malnutrition Universal Screening Tool.

Supplementary Table S3. Availability, location, and source of postoperative delirium assessment

Panel A. Quantifiable CAM assessment and delirium ascertainment data

| Assessment item | Overall cohort | GLIM-malnutrition group | Non-malnutrition group |
| --- | --- | --- | --- |
| Patients with at least one CAM assessment, n/N (%) | 432/495 (87.3) | 150/165 (90.9) | 282/330 (85.5) |
| Patients with at least one structured CAM record, n/N (%) | 301/495 (60.8) | 108/165 (65.5) | 193/330 (58.5) |
| CAM assessment documented in the PACU, n/N (%) | 214/495 (43.2) | 79/165 (47.9) | 135/330 (40.9) |
| CAM assessment documented in a general ward, n/N (%) | 389/495 (78.6) | 140/165 (84.8) | 249/330 (75.5) |
| CAM-ICU assessment documented, n/N (%) | 75/495 (15.2) | 28/165 (17.0) | 47/330 (14.2) |
| POD identified by a positive CAM or CAM-ICU assessment, n/POD cases (%) | 58/79 (73.4) | 33/42 (78.6) | 25/37 (67.6) |
| POD supported by a physician diagnosis, n/POD cases (%) | 52/79 (65.8) | 28/42 (66.7) | 24/37 (64.9) |
| POD supported by nursing records, n/POD cases (%) | 46/79 (58.2) | 25/42 (59.5) | 21/37 (56.8) |
| POD supported by specialist consultation, n/POD cases (%) | 31/79 (39.2) | 18/42 (42.9) | 13/37 (35.1) |
| Hypoactive delirium, n/POD cases (%) | 29/79 (36.7) | 18/42 (42.9) | 11/37 (29.7) |
| Hyperactive delirium, n/POD cases (%) | 21/79 (26.6) | 10/42 (23.8) | 11/37 (29.7) |
| Mixed delirium, n/POD cases (%) | 11/79 (13.9) | 7/42 (16.7) | 4/37 (10.8) |
| Delirium subtype unavailable, n/POD cases (%) | 18/79 (22.8) | 7/42 (16.7) | 11/37 (29.7) |

Panel B. Institutional delirium assessment procedures

| Assessment characteristic | Documentation |
| --- | --- |
| Routine CAM protocol | CAM was available for postoperative delirium assessment, but a mandatory fixed-frequency protocol for every eligible patient was not consistently documented throughout the study period. |
| CAM assessment personnel | Assessments were documented by trained ward nurses, attending physicians, anesthesiology personnel, and ICU staff, depending on the clinical location. |
| Assessment frequency | Assessments were performed during routine postoperative observation and whenever acute cognitive or behavioral change was suspected. Exact daily assessment frequency was not consistently available retrospectively. |
| Assessment locations | Post-anesthesia care unit, general surgical ward, and intensive care unit. |
| Structured CAM field | Available in part of the electronic medical record system, but not uniformly completed for all patients. |
| CAM criteria retrospectively applied to narrative records | No. Narrative records were used to identify documented diagnoses and supporting clinical descriptions, but the CAM algorithm was not retrospectively reconstructed when a formal CAM assessment was absent. |
| Determination of first POD episode | The earliest date and time of a positive CAM assessment or documented physician diagnosis was used. |
| Delirium subtype | Extracted when explicitly documented. Subtype was categorized as unavailable when the record did not permit reliable classification. |

Abbreviations: CAM, Confusion Assessment Method; CAM-ICU, Confusion Assessment Method for the Intensive Care Unit; PACU, post-anesthesia care unit; POD, postoperative delirium; GLIM, Global Leadership Initiative on Malnutrition; ICU, intensive care unit.

Supplementary Table S4. Fixed postoperative observation windows and alternative postoperative delirium definitions

| Outcome definition or restricted cohort | Eligible patients | POD events | Adjusted OR for GLIM-defined malnutrition | 95% CI | P value |
| --- | --- | --- | --- | --- | --- |
| POD during the entire hospitalization | 495 | 79 | 2.36 | 1.36–4.10 | 0.002 |
| POD within 48 postoperative hours | 495 | 45 | 3.01 | 1.52–5.96 | 0.002 |
| POD within 72 postoperative hours | 495 | 55 | 2.74 | 1.49–5.04 | 0.001 |
| POD from surgery through postoperative day 5 | 495 | 71 | 2.48 | 1.40–4.41 | 0.002 |
| Restricted to patients with a postoperative hospital stay of at least 48 hours | 483 | 79 | 2.34 | 1.34–4.08 | 0.003 |
| Restricted to patients with a postoperative hospital stay of at least 72 hours | 466 | 79 | 2.31 | 1.31–4.07 | 0.004 |
| Restricted to patients with a postoperative hospital stay of at least 5 days | 430 | 76 | 2.29 | 1.28–4.10 | 0.005 |
| Excluding POD first documented after postoperative day 5 | 495 | 71 | 2.48 | 1.40–4.41 | 0.002 |
| Restricted to structured CAM-positive POD | 495 | 58 | 2.42 | 1.31–4.46 | 0.005 |
| Restricted to physician-confirmed POD | 495 | 52 | 2.31 | 1.22–4.37 | 0.010 |
| Restricted to CAM-positive or specialist-confirmed POD | 495 | 66 | 2.39 | 1.33–4.28 | 0.004 |
| Excluding nonspecific nursing-record-only POD cases | 495 | 72 | 2.33 | 1.31–4.15 | 0.004 |
| Excluding periods of deep sedation or mechanical ventilation that precluded valid assessment | 481 | 74 | 2.29 | 1.29–4.08 | 0.005 |

Abbreviations: GLIM, Global Leadership Initiative on Malnutrition; POD, postoperative delirium; CAM, Confusion Assessment Method; OR, odds ratio; CI, confidence interval.

Supplementary Table S5. Availability of preoperative cognition, frailty, functional status, and muscle-related assessments

| Variable | Assessment method | Available, n/N (%) | Missing, n/N (%) | Used in GLIM diagnosis | Included in regression model |
| --- | --- | --- | --- | --- | --- |
| Mini-Mental State Examination | Formal MMSE score in the preoperative record | 26/495 (5.3) | 469/495 (94.7) | No | No |
| Montreal Cognitive Assessment | Formal MoCA score in the preoperative record | 12/495 (2.4) | 483/495 (97.6) | No | No |
| Mini-Cog | Formal Mini-Cog assessment | 9/495 (1.8) | 486/495 (98.2) | No | No |
| Other standardized cognitive screening | Any validated cognitive screening tool other than MMSE, MoCA, or Mini-Cog | 6/495 (1.2) | 489/495 (98.8) | No | No |
| Documented mild cognitive impairment | Review of diagnoses and preoperative medical history | 495/495 (100.0) | 0/495 (0.0) | No | No |
| Documented dementia or severe neurocognitive disorder | Review of diagnoses and preoperative medical history | 495/495 (100.0) | 0/495 (0.0) | No | No. Patients meeting the exclusion criterion were excluded before matching |
| Clinical Frailty Scale | Formal Clinical Frailty Scale score | 18/495 (3.6) | 477/495 (96.4) | No | No |
| FRAIL scale or another validated frailty instrument | Formal validated frailty score | 0/495 (0.0) | 495/495 (100.0) | No | No |
| Barthel Index or activities of daily living | Formal Barthel Index or ADL score | 74/495 (14.9) | 421/495 (85.1) | No | No |
| Instrumental activities of daily living | Formal IADL assessment | 11/495 (2.2) | 484/495 (97.8) | No | No |
| CT-derived skeletal muscle index | Skeletal muscle area normalized to height using a predefined CT level and threshold | 0/495 (0.0) | 495/495 (100.0) | No | No |
| Bioelectrical impedance-derived muscle mass | Appendicular skeletal muscle mass or skeletal muscle index measured by BIA | 0/495 (0.0) | 495/495 (100.0) | No | No |
| DXA-derived lean mass | Appendicular lean mass or lean-mass index measured by DXA | 0/495 (0.0) | 495/495 (100.0) | No | No |
| Calf circumference | Standardized preoperative calf-circumference measurement | 0/495 (0.0) | 495/495 (100.0) | No | No |
| Handgrip strength | Standardized dynamometer assessment | 0/495 (0.0) | 495/495 (100.0) | No | No |

Abbreviations: MMSE, Mini-Mental State Examination; MoCA, Montreal Cognitive Assessment; ADL, activities of daily living; IADL, instrumental activities of daily living; CT, computed tomography; BIA, bioelectrical impedance analysis; DXA, dual-energy X-ray absorptiometry; GLIM, Global Leadership Initiative on Malnutrition.

Supplementary Table S6. Sequential covariate adjustment and alternative matched regression analyses for GLIM-defined malnutrition

| Model | Regression method | Covariates included | Adjusted OR for GLIM-defined malnutrition | 95% CI | P value |
| --- | --- | --- | --- | --- | --- |
| Model 1 | Conditional logistic regression, with matched-set identifier as the stratum | Matched-set structure and GLIM-defined malnutrition | 2.74 | 1.65–4.54 | <0.001 |
| Model 2 | Conditional logistic regression, with matched-set identifier as the stratum | Model 1 plus age and sex | 2.62 | 1.56–4.39 | <0.001 |
| Model 3 | Conditional logistic regression, with matched-set identifier as the stratum | Model 2 plus chronic kidney disease and history of cerebrovascular disease | 2.53 | 1.49–4.29 | 0.001 |
| Model 4 | Conditional logistic regression, with matched-set identifier as the stratum | Model 3 plus surgical category and ASA class III–IV | 2.46 | 1.43–4.23 | 0.001 |
| Model 5 | Conditional logistic regression, with matched-set identifier as the stratum | Model 4 plus anesthesia duration per 10-minute increase and intraoperative blood transfusion | 2.40 | 1.38–4.16 | 0.002 |
| Model 6, primary fully adjusted model | Conditional logistic regression, with matched-set identifier as the stratum | Model 5 plus immediate postoperative ICU admission and C-reactive protein per 1-mg/L increase | 2.36 | 1.36–4.10 | 0.002 |
| Alternative model 1 | GEE logistic regression with matched-set identifier as the cluster and robust standard errors | Same covariates as Model 6 | 2.39 | 1.40–4.08 | 0.001 |
| Alternative model 2 | Logistic regression with matched-set cluster-robust standard errors | Same covariates as Model 6 | 2.37 | 1.38–4.07 | 0.002 |
| Operative-duration model | Conditional logistic regression, with matched-set identifier as the stratum | Same covariates as Model 6, with operative duration replacing anesthesia duration | 2.34 | 1.35–4.05 | 0.002 |
| Cognitive-status adjustment | Not estimated | Formal cognitive data were insufficient and nonuniform | Not estimable | Not estimable | Not estimable |
| Frailty adjustment | Not estimated | Formal frailty data were insufficient and nonuniform | Not estimable | Not estimable | Not estimable |

Abbreviations: GLIM, Global Leadership Initiative on Malnutrition; OR, odds ratio; CI, confidence interval; ASA, American Society of Anesthesiologists; ICU, intensive care unit; GEE, generalized estimating equations.
